# Numerical simulation and diagnosis geotechnical parameters of historical buildings in Najran City, Kingdom of Saudi Arabia

**DOI:** 10.1038/s41598-023-43959-1

**Published:** 2023-10-08

**Authors:** Gamil M. S. Abdullah, Ahmed Abd El-Aal, Ahmed E. Radwan, Hezam Al-Awah

**Affiliations:** 1 Civil Engineering Department, College of Engineering, Najran University, Najran, Kingdom of Saudi Arabia; 2 Faculty of Geography and Geology, Institute of Geological Sciences, Jagiellonian University, Gronostajowa 3a, 30-387 Kraków, Poland; 3 Geology Program, Department of Chemistry and Earth Sciences, College of Arts and Sciences, Qatar University, P.O. Box 2713, Doha, Qatar

**Keywords:** Engineering, Materials science, Physics, Environmental sciences, Environmental social sciences

## Abstract

This research aims to assess geoenvironmental risks and identify the primary deterioration drivers in ancient buildings in Najran City, utilizing various analytical tools to help make informed judgments. The samples extruded from historical buildings were examined using field inspection, experimental data, SEM, EDX, and XRD analyses, in addition to lab and field observations and meteorological data. The dissolution of clay minerals and salt crystallization are the key contributors to the degradation and cracking of historical buildings in Najran City, according to lab and field observations. When the daytime high temperature surpasses 44 °C, wind erosion and humidity might cause continuous wetting–drying cycles on the investigated building surfaces. Test results indicated that the average unconfined compressive strength of the extruded earthen wall samples was 2 MPa and the water absorption was within the upper allowed limit (i.e., 15%). A finite element model of a typical earthen historical building was developed using PLAXIS 3D software to assess the behavior and nonlinear response of the silty sand soil layer underlying the building and the earthen historical buildings themselves using a plastic material model. The field observations confirm the results of the simulation, which clearly explained the failure mechanism. The integrated geotechnical and numerical simulations could provide insights for assessing geoenvironmental risks, identify the primary deterioration drivers in ancient buildings, and provide an understanding of material qualities and failure causes not only in the studied area but in other similar regions elsewhere.

## Introduction

**Figure 1:**
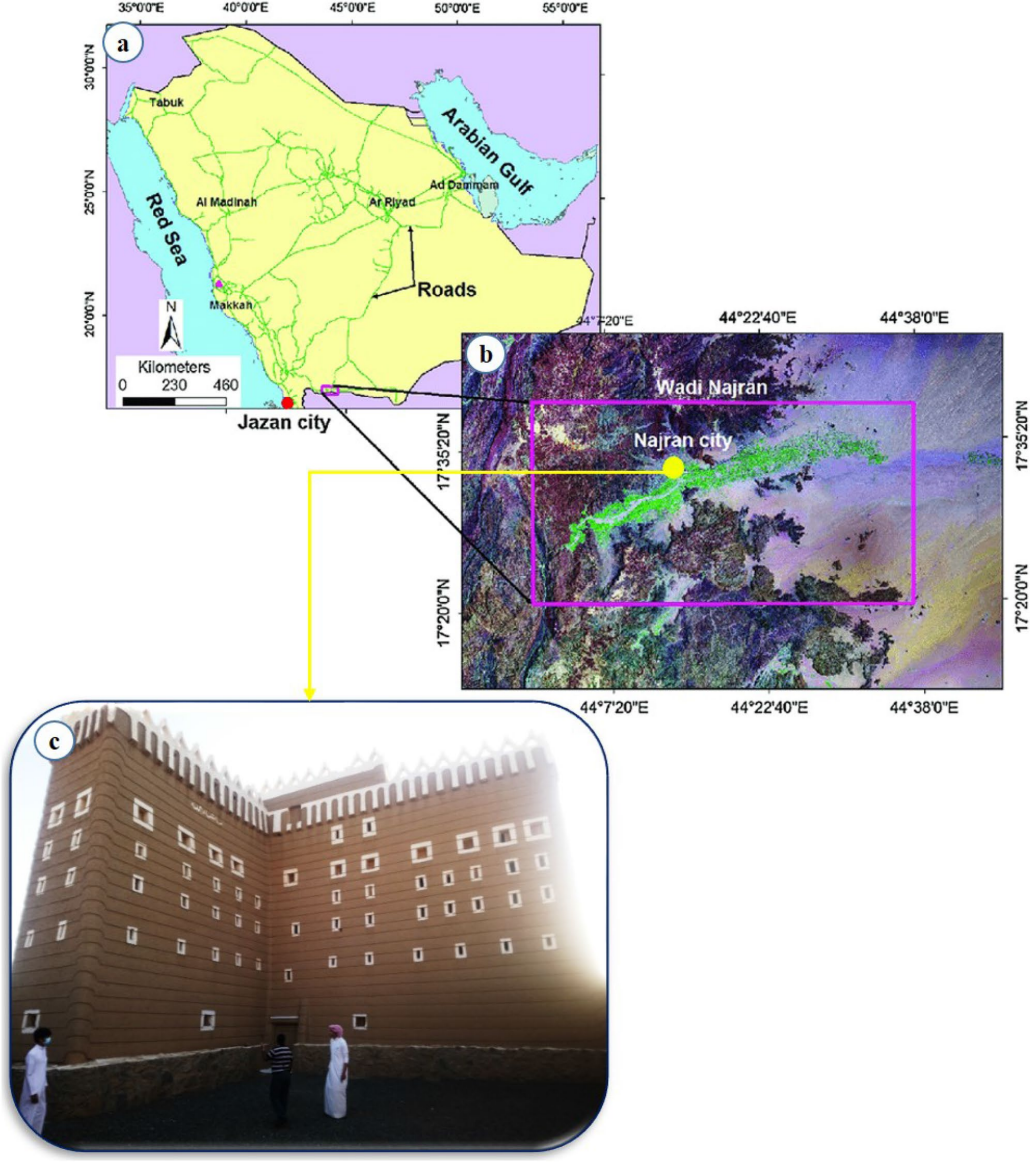
Location of the study area; note (**a**, **b**) are drawn by ARCGIS 2018 (https://www.arcgis.com/apps/View/index.html?appid=d6f0c301a4d5486fa5af281ef7bd0534); and (**c**) site photos illustrate the site under investigation taken by the author's digital camera.

Najran town, in southwestern Saudi Arabia near the Yemeni border, is the provincial capital of the Najran area. With a population that has risen from 47,500 in 1974 to 90,983 in 1992, then from 246,880 in 2004 to 505,652 in 2010, Najran is a new town and one of the Kingdom's fastest-growing cities. In the Najran region in southern Saudi Arabia, the research area is located between longitudes of 45°1′15′′ and 45°09′ E and latitudes of 19°01′′ and 19°5′30′′ N. Najran, located along Wadi Najran, is a key administrative and economic center in the region^1–4^. The Najran basin can be found in Saudi Arabia's far southwest (KSA). The arid Empty Quarter lies to the east, the Asir Region lies to the west, Al-Riyadh City lies to the north, and the Republic of Yemen lies to the south (Fig. 1a, b). Najran is a big area with a population of over 450,000 people and covers 359.9 km^2^. The basin under consideration is analogous to other basins in Saudi Arabia, which have had significant government and private sector development over the last 30 years. Several archeological sites are in the study area, but the Wadi Najran plain is a primarily agricultural area^5^. The most prominent governorates in the Najran region are Habuna, Yadamh, Thar, and Dhahran Al-Janoub. The mining sector and the use of stone as a building material have exploded in recent years as part of Saudi Arabia's 2030 goal. The mining industry is currently regarded as the country's third pillar and primary source of revenue. The most common uses for natural stone are flooring and decoration. In general, the Najran area is significant because the minerals and rocks found there contribute significantly to the region's national economy and have significant industrial potential, as well as some tourist sites. The most prevalent and widespread rocks and red clay include Najran granite, Wajid red sandstone, and basaltic rocks, with sand dunes being the most common deposits^6^. The historical buildings in the Najran area have importance for society and need special attention to prevent degradation and understand their characteristics. The main source of degradation in historical buildings is natural weathering processes (physical and chemical). In addition to man-made damage, they have resulted mostly through alternate methods of changing some climate factors, including temperature, relative humidity, and wind blowing. The historical building outcrop has been damaged due to alternate heating/cooling cycles caused by the type of clayey composition and different climatic variables, which explains the cracking phenomenon on the historical building under investigation.

The main objective of this work is to illustrate the impacts of weathering processes (physical and chemical) as the principal cause of degradation in historical buildings in the Najran area, as well as the impact of alternative mechanisms of certain climatic variables, especially temperature, relative humidity, and wind blowing. This research investigates the distribution of historical buildings in Najran, as well as providing a mineralogical analysis to investigate old buildings in Najran City and analyze geoenvironmental risks. Also, to identify the key deterioration drivers of the old buildings. This project will gather representative samples from various types of muddy and clayey buildings for visual and laboratory analyses in order to learn more about the geologic setting and distribution of historical buildings in Najran. These studies were carried out to determine the physical characterization of historical buildings in order to determine the quality of the collected samples, as well as other factors, including the physical characteristics of the studied sample units, chemical analysis in order to identify the formation of different types of samples, and the environmental impact of the studied samples. Furthermore, the purpose of this study is to determine which minerals have the greatest impact on the physical, mechanical, and water absorption of clay samples. Water absorption and X-ray diffraction (XRD) chemical analysis were all evaluated as part of the current study. Moreover, a finite element modeling of a typical earthen historical building was developed using PLAXIS 3D software to assess the behavior and nonlinear response of the earthen historical building itself and the underlying silty sand soil layer as well. This research is considered the first to deal with the study of historical and archaeological buildings in the city of Najran, which is considered one of the oldest historical cities in the Kingdom of Saudi Arabia and contains many historical buildings and castles, which are given utmost importance by the government, and it is an initial step for further studies in the same field.

### Study area and geological setting

The research area is located on the Arabian Shield's southeast side. In the Arabian Shield, a complex succession of Pre-Cambrian basement rocks is formed by new metamorphosed, interlayered volcanic, and ancient metamorphosed sedimentary rocks. Locally, igneous rocks ranging in age from the Pre-Cambrian to the Cambrian intrude on them, ranging from gabbro to syenite. Volcanic rocks in the area range in texture from andesite to rhyolite, aggregation to thick, massive flows, and lithic tuff. Sandstone, shale, conglomerate, and limestone, in various forms, are commonly interlayered with sedimentary strata. The basement rocks are not encumbered by unconsolidated alluvial, aeolian sandstone, or Cambro-Ordovician Wajid sandstone, according to^6^ by (Lower Red Unit). Most of Najran's western portion has been eroded, and the Wajid sandstone is now only found on isolated buttes where the basement has been trapped.

## Research methodology 

**Figure 2:**
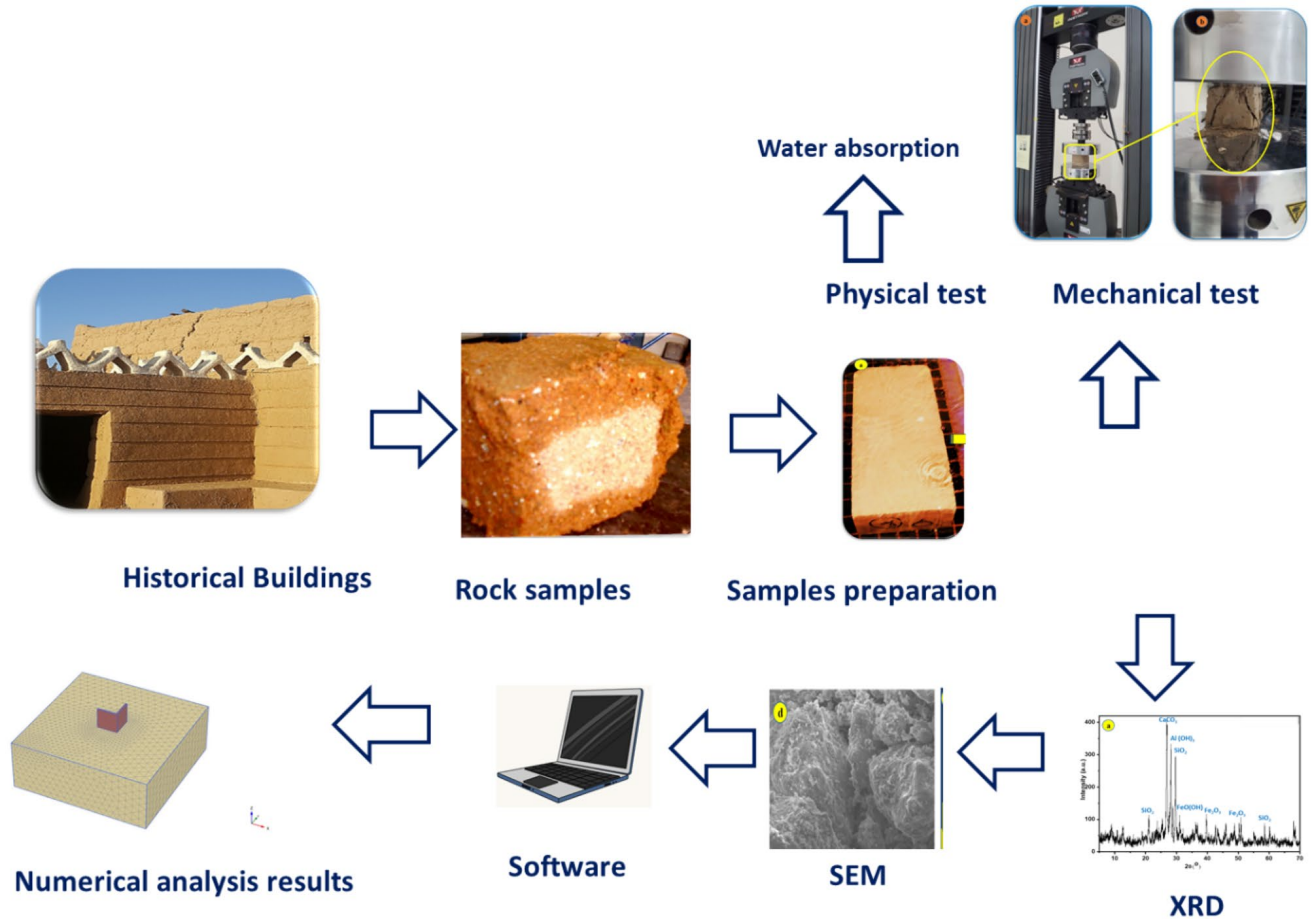
A detailed flowchart to explain the methodology and experimental tests of the present work.

The harmful environmental variables that describe and analyze the degradation of historical structures in Najran City are temperature and humidity fluctuations, day and night, as well as wind erosion. Samples were chosen to depict the true level of historic building degradation in Najran City. This research aims to investigate the heritage buildings in Najran City, Kingdom of Saudi Arabia, through the diagnosis of their geotechnical conditions using a series of field and lab tests. In addition, conducting a finite element simulation to forecast their engineering behavior and main causes of potential collapse. The methodology used to accomplish this target involves four phases: (i) the gathering and classification of construction and building ingredients of heritage buildings; (ii) performing field and laboratory investigation tasks; (iii) studying the outcomes of laboratory experiments and matching them with field test results; (iv) conducting finite element modeling to forecast the engineering performance of earthen historical buildings; and (v) investigating the environmental impacts. To assess the presence of secondary phases that could explain why the earthen historical buildings were damaged, these samples were submitted to mineralogical identification, petrographic inspection, and chemical analysis^7^. SEM equipped with EDX (JEOL/EO, JSM-6380 device) was used to characterize the degraded flakes from the collected samples. Field observations will aid in explaining the weathering mechanism's impact on historical structures. Figure 2 presents the research methodology followed in this study.

### Geotechnical conditions

Based on topographic maps, the research region can be divided into three geomorphological units: (i) high-mountain areas around the research area; (ii) flood plain areas along the Wadi of Najran; and (iii) sand dunes along the Empty Quarter's borders. The majority of the rocks in the Najran area are igneous, with some stratified Wajid sandstone and Tertiary bedrock thrown in for good measure^6^. Quaternary surficial elements include alluvial deposits from Wadi Najran and sand dunes, especially between Wadi Najran and the Empty Quarter.

**Figure 3:**
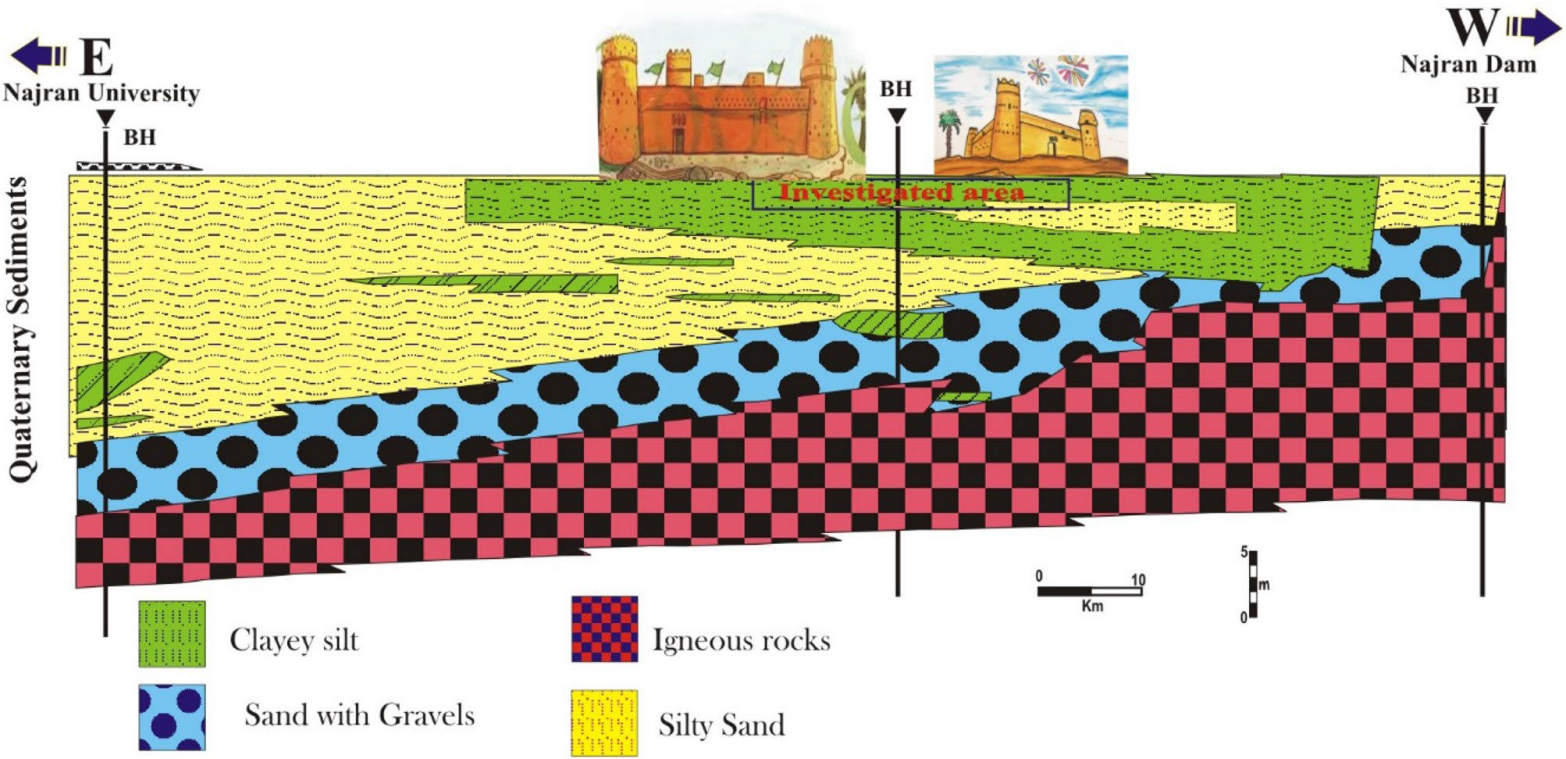
E–W profile along the area of study area showing the subsurface layers of foundation beds of the historical building.

The subsurface exploration of the Najran city area consists of multi layers of thick silty sand and dune sand, especially in the east direction. A clayey silt layer mixed with variable sand is extended in the middle and west directions (Fig. 3). These layers overlay layers of sand with gravel and igneous rocks, as shown in Fig. 3. The subsurface water does not appear within the top layers that extend to a depth of about 10 to 15 m. The geotechnical examinations were executed on the collected soil specimens. Information was gathered from multiple boreholes, distributed along the area of study. The results of laboratory tests were for sieve analysis (ASTM D422), liquid limit (LL) (ASTM D423), plastic limit (PL) (ASTM D424), and direct shear tests (ASTM D3080). The geotechnical tests were conducted in the soil lab of the College of Engineering at Najran University.

**Figure 4:**
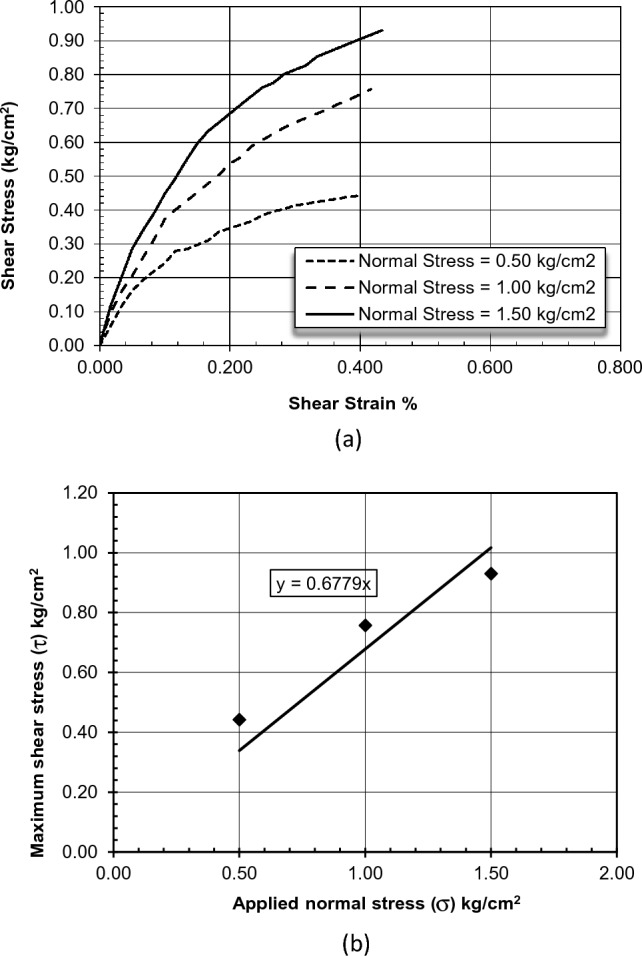
Direct shear test results (**a**) shear strain versus shear stress (**b**) normal stress versus shear stress.

For Sandy Layers, the results of lab examinations show that the LL = 20, the PL = 0, the natural unit weight γ = 18 kN/m^3^, and the saturated unit weight γ_sat_ = 20 kN/m^3^. The modulus of elasticity ranges from 20,000 to 50,000 KN/m^2^, and Poisson’s ratio equals 0.30. Furthermore, to get the shear strength parameters of these silty sand layers, a direct shear box test was conducted. The test was done under (ASTM D3080) on many disturbed samples taken from different boreholes located in the area of study, and the results for one test as an example are presented in Fig. 4 below. The results of the direct shear box test showed that the cohesion (c) parameter value of the soil was zero, whereas the angle of internal friction (ɸ) parameter was in the range between 28° and 37°.

The modulus of elasticity of the soils was determined based on the standard penetration test results conducted at the research sites by using correlation equations. However, for the earth walls, the modulus of elasticity was calculated based on the unconfined compressive strength test results. The results of the laboratory test of the soil layer are inconsistent with the results reported in the previous studies^1–5^.

## Physical and mechanical properties of building materials

### Field observation 

As a result of the rains, humid stains can be seen in the vicinity of old buildings. The buildings then developed vertical cracks on the right side. There are also clay droppings and degraded areas at the bottom of the second side of the old buildings. Water weathering is visible as erosion and spots within the claystone on the external floors of the buildings. The western façade, which is in the shade almost all day, is believed to be the most badly damaged in terms of separation and cracking of claystone blocks from their bottoms, as well as the formation of cracks in the higher areas.

### Micro-tests

#### XRD

**Figure 5:**
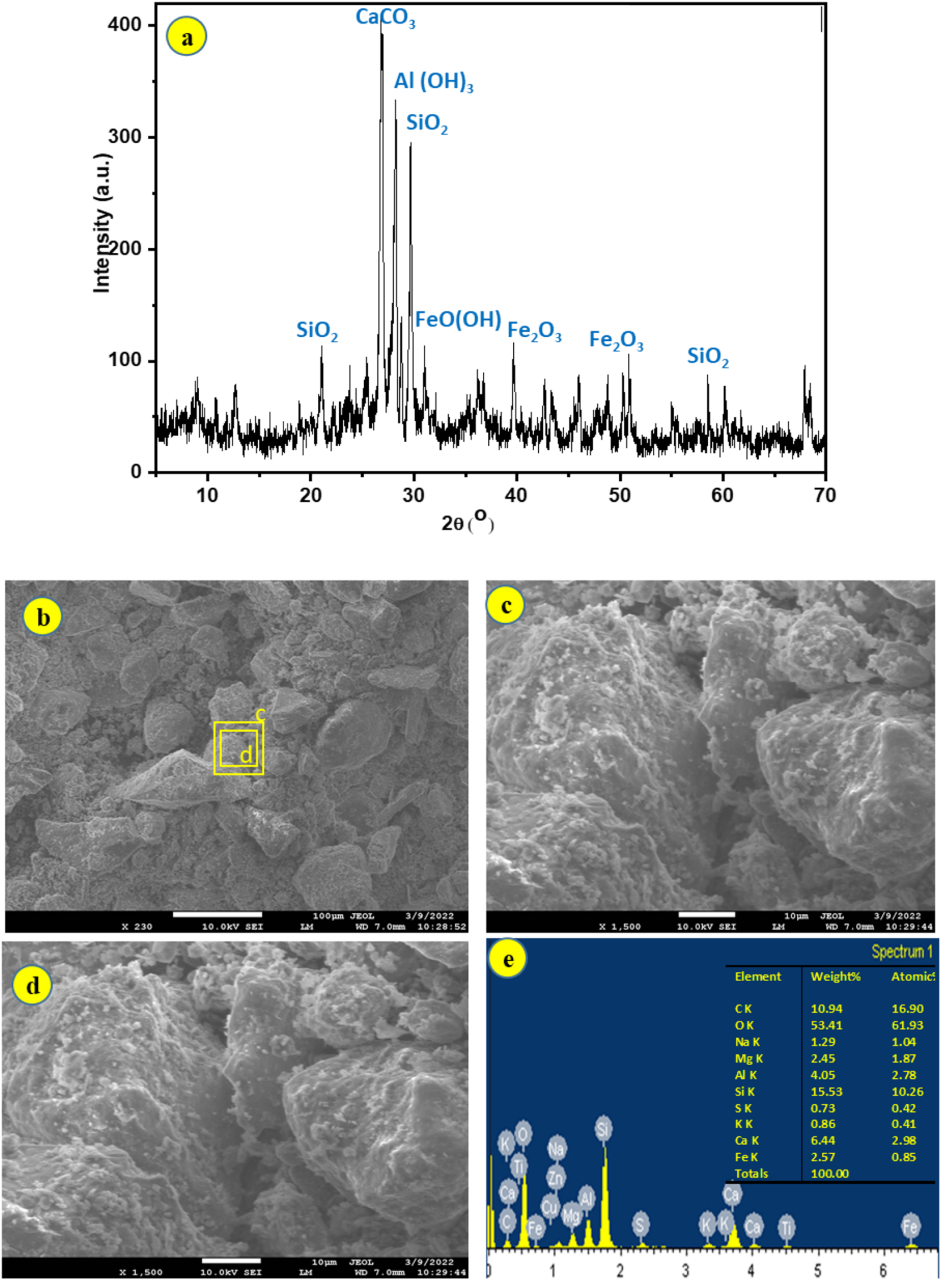
(**a**) Different XRD patterns of clay samples; (**b–d**) SEM images of claystone mainly (Illite); and (**e**) EDX for clay spectra, which indicate the composition of samples under investigation.

The mineral composition of the tested samples was determined using the x-ray diffraction powder technique (XRD); XRD results reveal that kaolinite is the predominant ingredient (approximately 90.28–93.41%). Illite makes up the clay component (Fig. 5a). Some samples revealed traces of halite and goethite minerals.

#### SEM

Under SEM examination of a sample representing the main buildings, thin coatings of booklets and salt crystals were seen on parts of the Illite and pores. The surface deterioration and disintegration of Illite can be seen (Fig. 5b–d). The findings indicate that Illite is the major ingredient. The identity of Illite is confirmed by EDX analysis, which shows roughly similar peak heights of the mineral and the feldspars constituents, which comprise about 53.4% by weight of the samples (Fig. 5e).

### Macro-tests

Samples taken from historical building were tested to assess their compressive strength and water absorption.

#### Unconfined compressive strength (UCS) test

**Figure 6:**
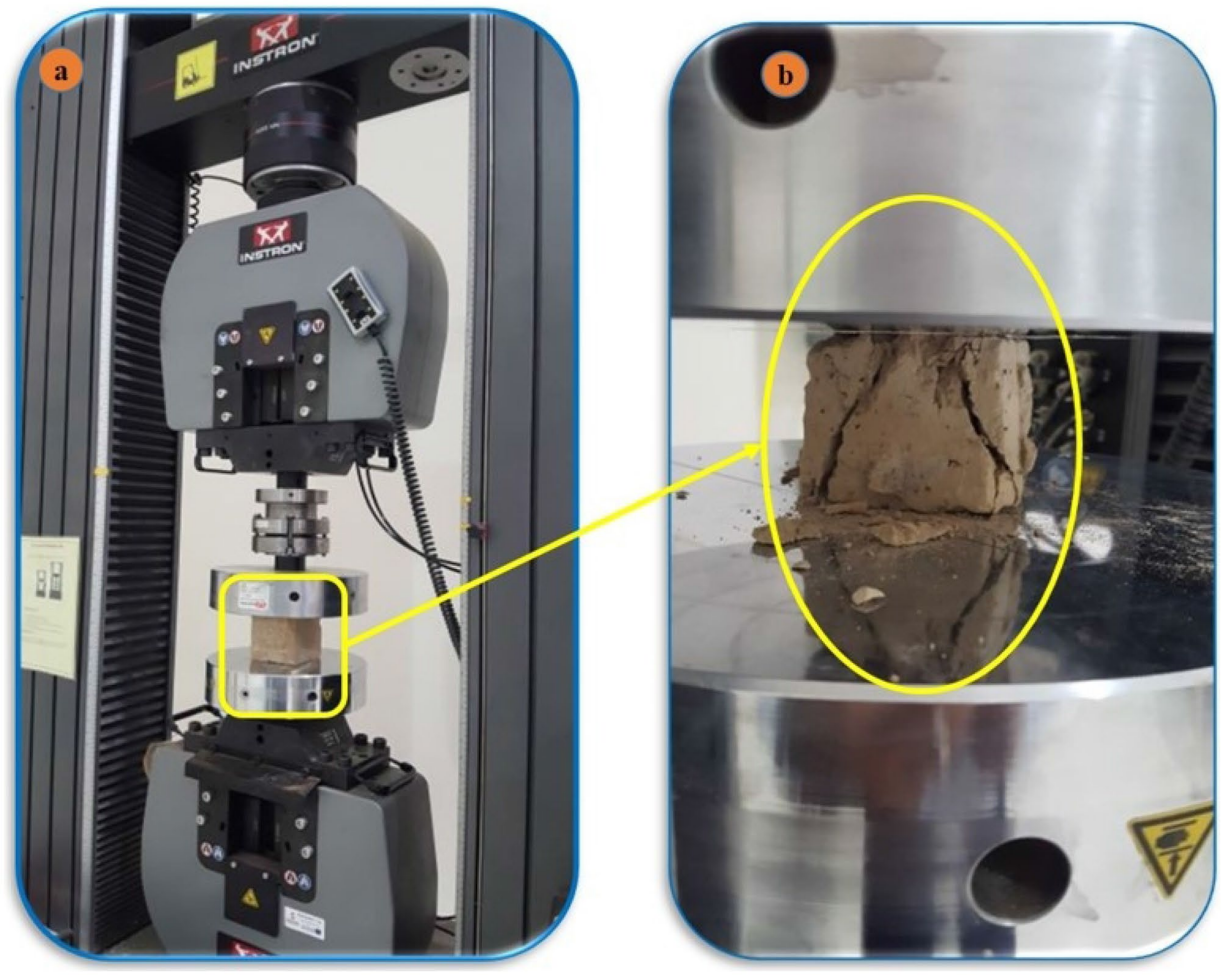
Compressive strength test of prepared cubed from historical buildings (**a**) test setup, (**b**) sample after failure and loading rate.

At the beginning and during the field visit to the earthen historical buildings in Najran city, non-destructive testing was also conducted by the Schmidt hammer to assess the UCS of the walls of the buildings. Thereafter, some samples were retrieved from the building for lab tests. Figure 6 shows the strengths of the earthen wall units measured by an unconfined compression test machine.

A concrete cube test apparatus or an equivalent instrument was used to test individual specimens. The load was gradually increased until it failed. The maximum load and original gross cross-sectional area were used to calculate each specimen's compressive strength^8,9^. Dry specimens were tested in an ambient or oven setting. In either case, the moisture content at the time of testing has a big impact on compressive strength^10,11^. According to the Center for Development of Enterprise (CDE) guidelines (African Standard), any randomly selected sample should be evaluated with a minimum of five samples. The test involves applying simple compression to a sample until it fails. The effect of a constantly rising pressure generated by compression was used to position and crush cubic specimens (5 × 5 × 5 cm) retrieved from block samples in the direction in which they were laid. After complete failure, crushing was contemplated.

The required specimen was centered in the middle of the compression plates. The loaded surface's geometric center is on the axis of the tested block (Fig. 6a). The machine was switched on, and block dimensions were placed. The load was started at a constant rate of 3 mm/min, with no abrupt shocks, at a steady speed. The pressing was continued until failure, and the compression machine was stopped automatically (Fig. 6b). The average strength results of five tested specimens were found to be 2.0 MPa.

#### Water absorption test

The water absorption test aimed to determine what percentage of the retrieved blocks absorbed moisture. The absorption test was carried out according to^12^. Blocks were cleaned, put in the oven to dry, and then removed to cool. A water tank was filled with water until it reached around 125 mm in height, which was suitable to cover the tested block. The tested earth blocks have dimensions of 290 mm long, 140 mm thick, and 90–100 mm high. The blocks were weighed, and after they were recorded, they were immersed immediately in the water tank. After that, they were put on adjacent wedges to let the water surround the tested blocks, and water was allowed to cover the block more than 25 mm above the top surface. The blocks were removed after 30 min, and the free water was allowed to seep. Using a piece of cloth, the blocks were wiped and then weighed. The process of removing the blocks from the water, wiping, and weighing did not take more than 2 min. This was to ensure that the right measurements were obtained.

**Figure 7:**
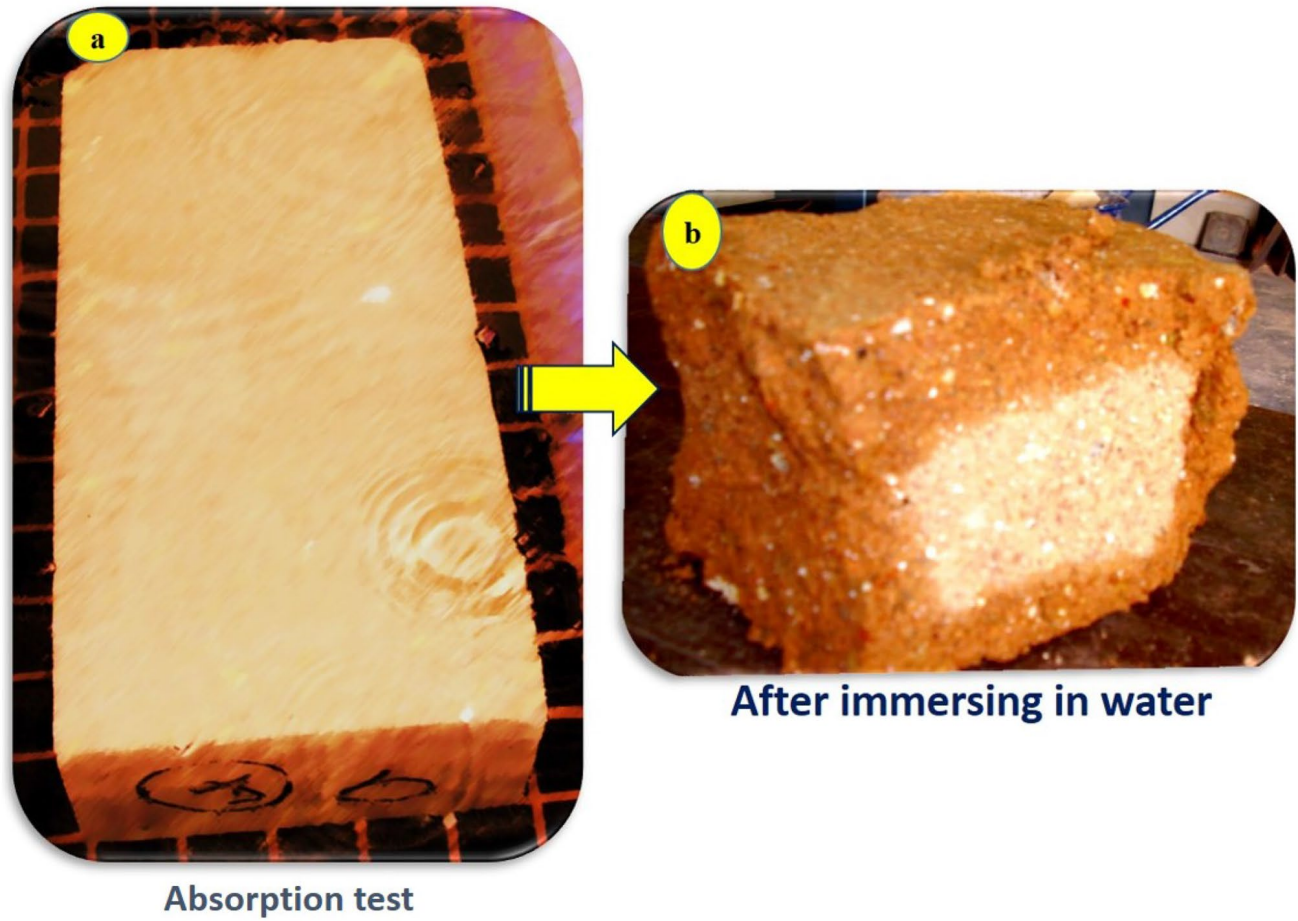
(**a**) Water absorption test of study samples; (**b**) section of sample after immersing in water to illustrate the ratio of water absorption.

An electronic weighing machine was used in this case, with an accuracy of 0.1 g. The percentage of moisture absorption by weight was calculated from the formula using the method derived from the British standard for determining water absorption,^12^. The absorbed water was determined using the following formula:

1 $$\frac{W1 - W0}{{W0}} \times 100$$

where W_1_ is the block weight immediately after testing and W_O_ is the weight of the dried block after oven drying and before immersing. The apparatus consists of an accurate weighing balance, a stop clock, and a water trough or tank with a capacity to hold up to five fully immersed blocks. The recommended maximum water absorption was 15%^13^. Houben and Ggillaud^13^ proposed that the maximum value of water absorption for blocks is 20%^14^. Figure 7a, b show the absorption test and a cross section of the specimen after the absorption test, respectively. Based on the absorption test results, the average water absorption of the five specimens was 15%.

## The architectural design of historical buildings

**Figure 8:**
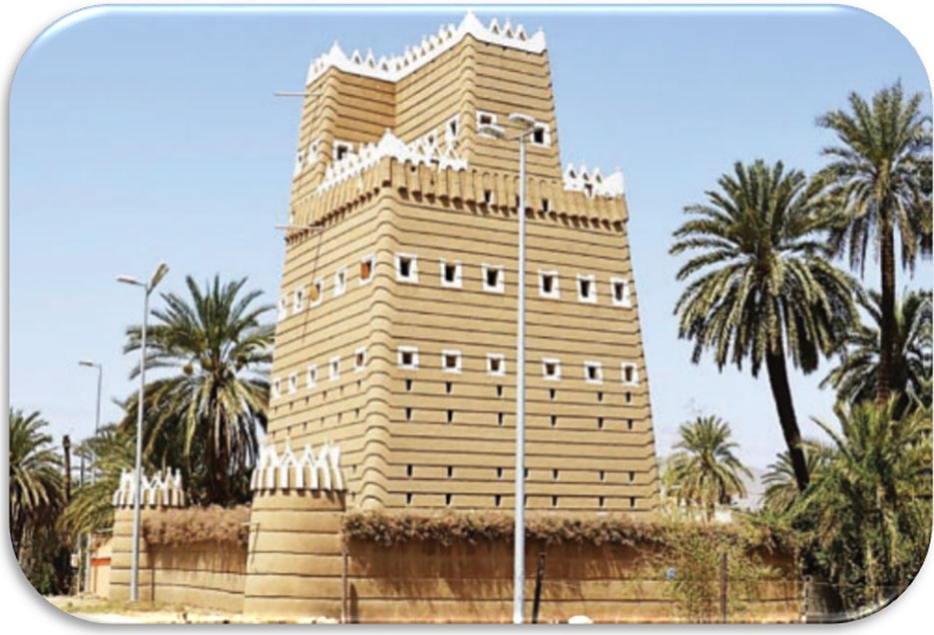
Shape of general mud cob building in the Najran Valley, after^5^.

Heritage buildings in Najran City commonly have a square or rectangular shape with three to four stories. The walls of these structures have approximately 50 cm of thickness and are constructed from local earthen materials using cob techniques^5^. Traditional structures in Najran are known for their earthen architecture legacy, and they offer a wide range of benefits as well as a beautiful variety of forms and external designs, as seen in Fig. 8. Najran's earthen building architecture is unique. Traditional "cob" techniques were used to construct four- to five-story mud houses. Mud cob construction has been a thriving regional industry in the Najran Valley for millennia, resulting in long-lasting and elegant structures, as shown in Fig. 8. Earthen construction in the Najran Valley has a long history of sustainable buildings built using local resources and cob technology.

## The current state of historical buildings

**Figure 9:**
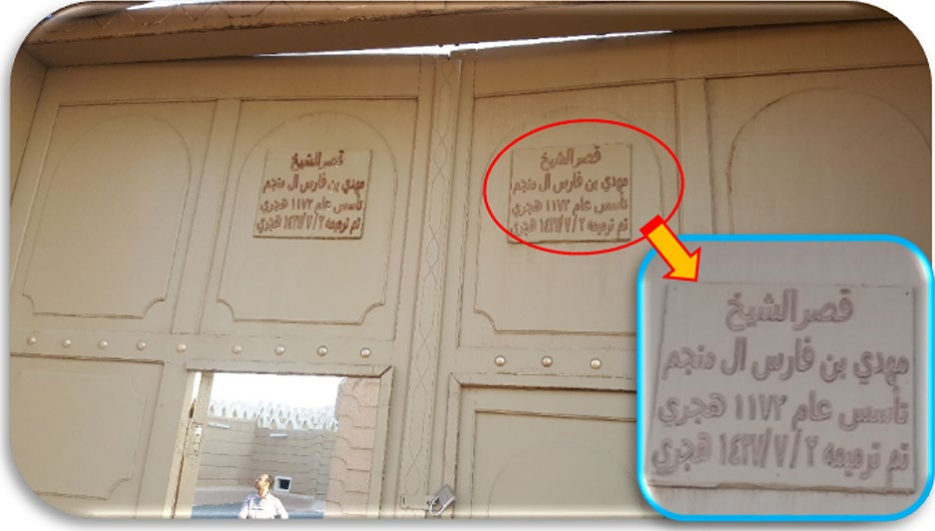
External gate of one historical palace, showing the date of its construction and renewal.

The earthen heritage buildings in Najran city are very old. Some of these buildings have been revered for hundreds of years, as seen in Fig. 9, which shows the date of construction and renovation of one historical palace. The date of construction was 1759 A.D. Due to the nature of the construction materials of these kinds of heritage buildings, the surrounding environmental conditions, such as intensive rainfall, have bad effects on the durability of these earthen materials. Moisture content is frequently the most important factor in the degradation of construction and building materials. Therefore, heritage buildings are frequently subjected to regular maintenance from local materials^5^.

Three key factors have been considered to identify and evaluate the damage to Najran's historical buildings: geological formation, environmental circumstances around the building, and man-made damage, which are addressed in the following sub-sections.

### Geological formation 

The historical building of Najran is located on a vast plain on the piedmont of Wadi Najran, a massive basaltic deposit in the valley's stream. The Najran claystone valley is grey to brown in color. Claystone normally has a fine texture; however, layers of pebbles and cracks are common in claystone. Because of these qualities, they are less suitable for rock cutting than finer, more homogeneous claystone. From a lithological standpoint, it's worth noting that claystones are not always resistant to weathering, and some faces are more prone to erosion than others.

### The surrounding environment

The summers in the Najran area are long, hot, clear, and arid, while the winters are short, dry, chilly, and mainly clear, according to meteorological statistics. From May 12 to September 19, the hot season lasts 4.2 months, with an average daily high temperature of over 94 °F (34.4 °C). July is the hottest month in Najran, with average highs of 99 °F (37.2 °C) and lows of 77 °F (25 °C) (weather spark 2020).

Because the dew point influences whether perspiration evaporates from the skin and chills the body, it is used to determine the level of humidity. It feels dryer when the dew point is lower and more humid when the dew point is higher. Because the dew point changes more slowly than the temperature, a humid day is frequently followed by a muggy night, even if the temperature drops at night.

The number of wet days in Najran does not change much during the year (i.e., those with greater than 0.04 inches of liquid or liquid equivalent precipitation). The frequency ranges from 0 to 4%, with an average value of 2%.

We distinguish between days with rain only, snow only, or a combination of the two types of precipitation. With an average of 1.1 days of rain per month in Najran, April is the month with the most rain days. According to this classification, rain alone is the most common form of precipitation throughout the year, with a peak likelihood of 4% on April 21.

**Figure 10:**
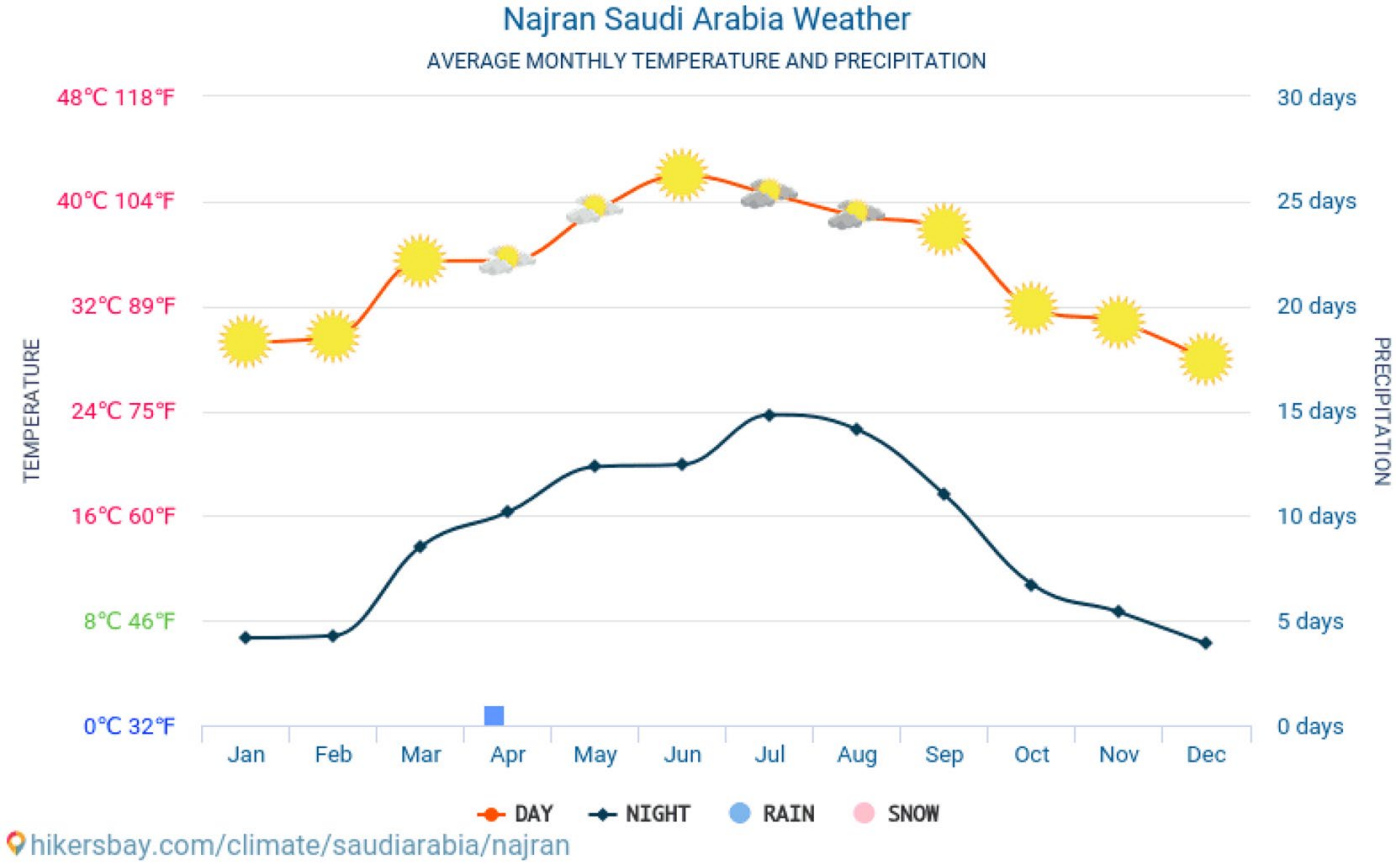
Average monthly temperature and precipitation of Najran City.

The sliding 31-day rainfall quantity at Najran does not vary significantly during the year, remaining within 0.1 to 1.1 in. Figure 10 shows the Average monthly temperature and precipitation of Najran City.

In Najran, the amount of time when the humidity comfort level is muggy, oppressive, or unpleasant does not fluctuate significantly throughout the year, remaining at a fairly constant 0%.

This section discusses the wide-area hourly average wind vector (speed and direction) at 10 m above the ground. Because of local geography and other factors, wind speed and direction fluctuate more than hourly averages at any given location, and instantaneous wind speed and direction vary more than hourly averages. The average hourly wind speed in Najran varies greatly by season throughout the year. The windier part of the year lasts 2.2 months, from June 20 to August 26, with average wind speeds of more than 7.8 miles per hour. With average hourly wind speeds of 9.1 miles per hour, July is the windiest month in Najran. The quieter season lasts 9.8 months, from August 26 to June 20. With an average hourly wind speed of 6.3 miles per hour, December is the calmest month in Najran. Rainfall and normal fluctuations in relative humidity (about 20% in the summer and 40% in the winter) cause the clay minerals in the stones to enlarge. Furthermore, the variety of mineral particles may produce stress as a result of temperature changes and the influence of humidity over time. Scaling occurs when thermal stress creates shear tension at grain boundaries and cracks^14^. Daily temperature, humidity, 12 h of daylight each year, and wind erosion from three directions can all contribute to the continual wetting–drying cycles on stone surfaces (west, north, and east). According to a previous study, natural weathering processes, both physical and chemical, are the principal source of ancient structural degradation^15,16^. They originated mostly because of various climate factors' different methods. Thermal shock (30.33 °C high 22.22 °C low in summer) and (high of 20.56 °C and low of 4.33 °C in winter) stretch and contract the claystone outcrop in different ways. According to^15,16^, this process is driven by high temperatures, which frequently disrupt construction by causing temperatures to climb during the day and decrease dramatically at night. The building heats up throughout the day and then shrinks at night due to the linear thermal expansion coefficients of the sandstone, causing the surface to retreat due to tension induced by size changes and pressures on the joining materials^16^. Finally, it produces continuous changes in the mineralogy's physical or chemical properties throughout time. Due to the large range in season quantities, where the greatest MR (4 mm) is recorded in April and thereafter MR is recorded for several months, precipitation levels are the key drivers of degradation (0.0 mm). As a result of blown clay abrasion, wind blowing is regarded as one of the reasons for building cracking, especially in the areas nearest to the ground floor. In reality, wind speed has a significant impact on wind erosion processes. The greater the ability of the wind to move coarse sand grains, the greater the rate of outcrop erosion.

### Damage caused by humans

In addition to damage produced by the weathering process, there is also harm inflicted by humans. Man-made damage in the old architecture of Najran includes spray paint patterns and scrapes made with sharp metal tools, as well as holes over the main door. The aforementioned components of the SCTA's damage have been restored using a variety of approaches.

## Thermal conductivity

Thermal conductivity is vital in material science, research, building insulation, and other industries where high operating temperatures are required^17,18^. According to prior research, clay has superior thermal insulation characteristics, indicating that it has a strong potential for usage as a building material, particularly in hot countries such as Malaysia^19^. Thermal conductivity (k) is the amount of heat that passes through homogeneous materials with a thickness of 10 mm to 20 mm and a diameter of more than 30mm. It is also the amount of heat that will flow through unit area in unit time when there is a temperature differential between the faces of materials of unit thickness. Thermal conductivity tests on clay and mortar were conducted as part of this research. According to certain studies, clay with lower thermal conductivity generates less conductive concrete. All the specimens in this test have a thickness of 20 mm and a diameter of 75 mm, and they were baked for 24 h at 80 °C before being tested. The testing will be done with a transient plane source; the specimen will be measured by recording the temperature rise of the source plan heating the sensor's surroundings. As a result, the result will be determined by the distribution and percentages of artificial lightweight aggregate present in the specimen. Mortar has a thermal conductivity of 1.74 W/mK, while clay has a thermal conductivity of 0.17 W/mK.

## Numerical analysis

The preservation of earthen structures constructed using cob techniques necessitates a thorough understanding of material qualities and failure causes. However, this knowledge is still scarce and dispersed throughout the literature. Numerical modeling of earthen constructions is also a useful tool for making decisions about their preservation.

Because there are vertical and horizontal mortar joints, the masonry is anisotropic. Two different methodologies have been employed to replicate such anisotropy: the micromodel,' or 'two-material approach,' and the macromodel, or 'equivalent-material approach'.

Since heritage earthen buildings are constructed in Najran City, there is something called cob construction that is different from masonry. The walls are considered one unit with no joints; in other words, the mortar used in the joints (if they exist) is made from the same mud materials as the wall units. Therefore, a macromodeling continuum approach is adopted. The earthen wall is treated as a homogeneous continuum medium in this approach.

Using a plastic material model, PLAXIS 3D V2020^20^ was used to examine the behavior and nonlinear response of a silty sand soil layer and an earthen historical building construction. PLAXIS is a finite element method (FEM) program that is available for purchase. PLAXIS defines soil behavior using soil models such as the Mohr–Coulomb Model, Hardening Soil Model, Soft Soil Creep Model, Jointed Rock Model, and Modified Cam–Clay Model. The Mohr–Coulomb Model was selected for earthen wall simulation since it is commonly used and does not require additional soil factors. Furthermore, it can also be used to simulate the behavior of the earthen historical building structures since these buildings are constructed using earthen materials such as clay or mixed clay and silt as per the specifications stated in the literature.

Young's modulus (E) and Poisson's ratio (v) for soil flexibility; Cohesion (c), friction angle (ϕ), and dilatancy (ψ) for soil shear behavior are the five parameters employed by the Linear-Elastic Perfectly-Plastic model (Mohr–Coulomb). At a first-order level, the Mohr–Coulomb model approximates soil or rock behavior. In a basic and applicable three-dimensional stress space model, the Mohr–Coulomb model represents the plastic conduct of earth soil, its submerged conduct, and related streams. This model performs better in terms of quality behavior. This model can be used to assess the stability of shallow foundations as well as soil issues. The dilatancy angle of the soil is used to define the Mohr–Coulomb flow rule. The Mohr–Coulomb model predicts continuous dilation; however, volumetric plastic strains on shearing in soft soils are compressive (negative dilation).

The model of hardening soil accessible in Plaxis 3D was used to model the nonlinear performance of the soil layer material. The hardening soil model is a hyperbolic strain—stress model with limit phases that are similar to Mohr–Coulomb's in terms of the angle of friction $\mathrm{\varnothing }$, cohesion $c$, and angle of dilatancy ψ. In the hardening model, there are three different inputs of stiffness to represent the dependency of soil stiffness on applied stress. These inputs are the triaxial stiffness (${E}_{50}$), the oedometer loading tangent stiffness (${E}_{oed}$), and the triaxial unloading stiffness (${E}_{ur}$). In addition to secant, oedometric, and unloading–reloading stiffness, the model is defined by extra parameters such as reference stress and a power factor (${p}^{ref}$, $m$).

The PLAXIS 3D v20 was used to perform numerical modeling of the plane strain. To eliminate mesh size effects, a fine precision mesh network was adopted. The generated number of nodes and elements is equal to 40,342 and 27,062, respectively. All the soil layers' geotechnical parameters used in the modeling and engineering characterization of the construction materials are listed in Table 1.

**Table 1:** Parameters used in the finite element modeling.

Parameter	Soil layer	Earthen wall
Unsaturated unit weight, γ_unsa_t (kN/m^3^)	18	21
Saturated unit weight, γ_sat_ (kN/m^3^)	19	22
Material model	Hardening soil	Mohr–Coulomb
Drainage type	drained	Undrained
Poisson’s ratio ν	0.3	0.35
Cohesion, C (kN/m^2^)	0	1000
Angle of friction, φ (°)	37	0
Angle of dilatancy, Ψ (°)	7	0
Modulus of elasticity, E (kN/m^2^)	–	300.0E3
${E}_{50}$ ref (KPa)	16.67E3	–
${E}_{oed}$ ref (KPa)	16.67E3	–
${E}_{ur}$ ref (KPa)	50E3	–
Power in stiffness laws, $m$	0.5	–
Unloading–reloading Poisson’s ratio, $\nu$	0.2	–
Interface reduction factor, ${R}_{inter}$	1	1

**Figure 11:**
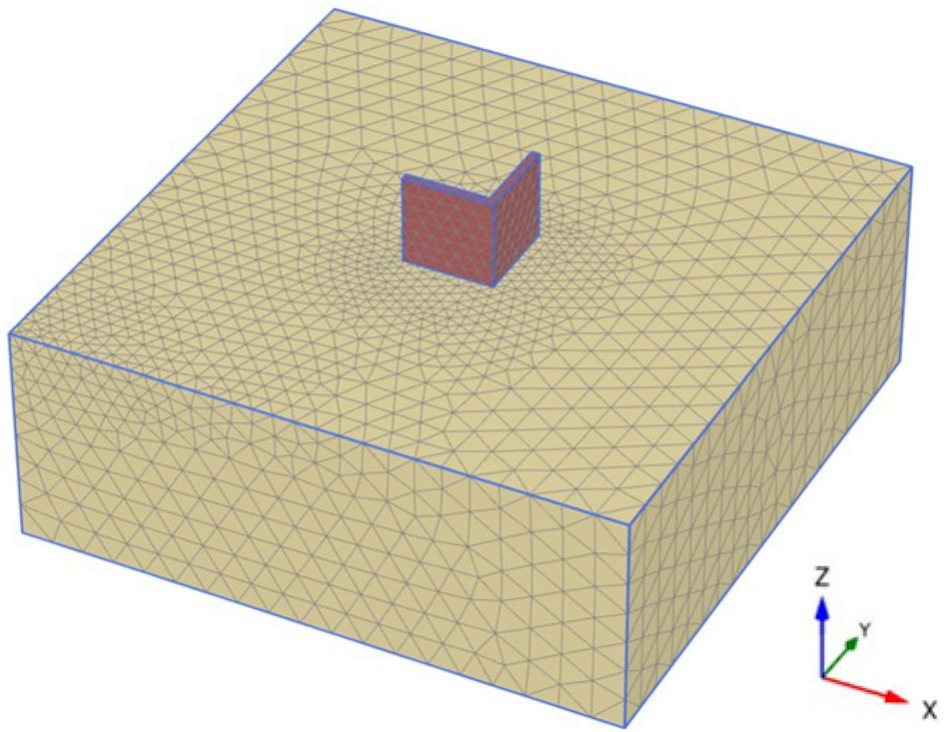
Model illustrating the geometry and analysis of the subsurface soil and heritage earthen structure.

Figure 11 shows the geometry of the adopted model of the subsurface soil and heritage earthen structure. The subsurface layer is a silty sand soil extending to 15 m, and no ground water was detected at this depth. As stated before, the heritage buildings in Najran city have a square or rectangular shape. Thus, to reduce the calculation time of the numerical simulation, corner parts of the earthen structure with a length of 6 m in each direction (x and y) and a thickness of 0.5 m were simulated, apart from the whole structure having dimensions of 12 m by 12 m. The height of the walls was taken as 6 m, representing three floors, where the height of each floor is approximately 2 m in the old building system. The dead and live loads were added to the walls as distributed loads. To avoid any undesirable effects of the model boundaries, the length of the model geometry in the horizontal direction was taken to be more than four times the length of the walls, and the vertical dimension of the model geometry was extended to be more than twice the height of the walls. The boundary conditions were fixed laterally in horizontal directions, while they were fixed vertically and horizontally in the base. The Mohr–Coulomb elastic-perfectly plastic constitutive model was adopted to simulate the earthen walls, which were assumed to be in an undrained state. However, the hardening soil model was used to simulate the soil layer below the wall since the modulus of elasticity changes at different loading stages. Hence, it is preferred to use the Hardening Soil model.

The initial stresses for the soil layers were generated using the K_o_ technique in Plaxis to bring the model into equilibrium. Following that, plastic calculations were used to activate the earthen walls in order to capture the response and replicate the construction process. The model was then examined under the dead and live loads as well as the self-weights of the walls. All the components were turned on at the same time, with the expectation that the walls would be built from the bottom up.

## Numerical analysis results

**Figure 12:**
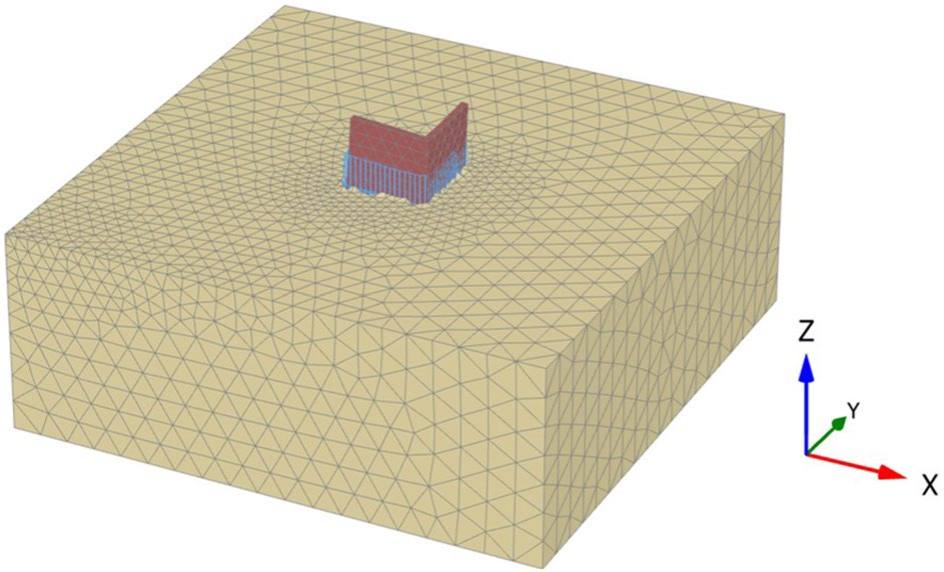
Schematic sketch of finite element discretization of the 3D model and the deformed mesh of historical buildings in the Najran area.

**Figure 13:**
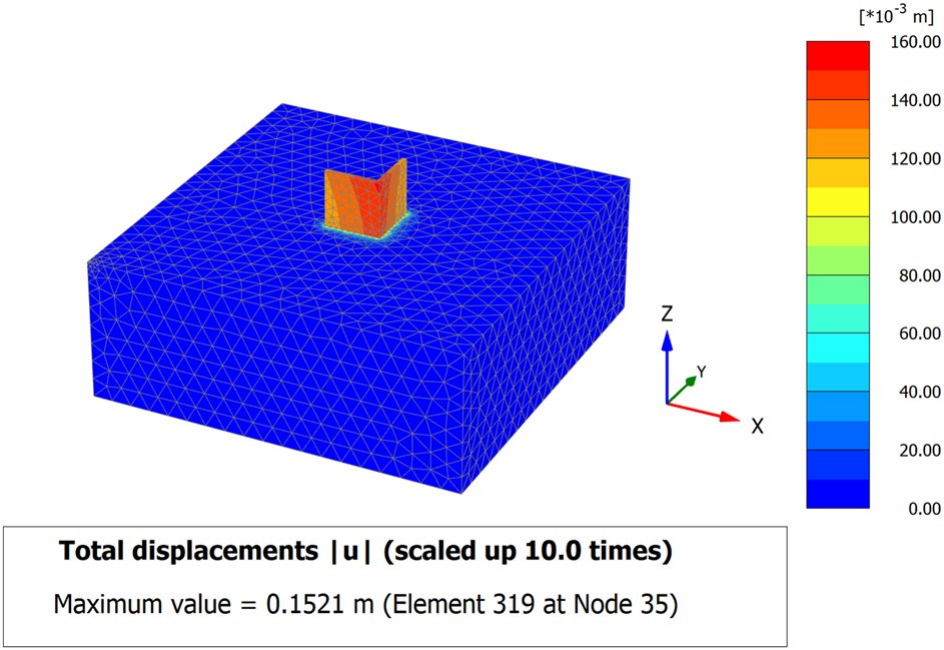
3D model of the total and maximum total displacement IuI of historical buildings in the Najran area.

**Figure 14:**
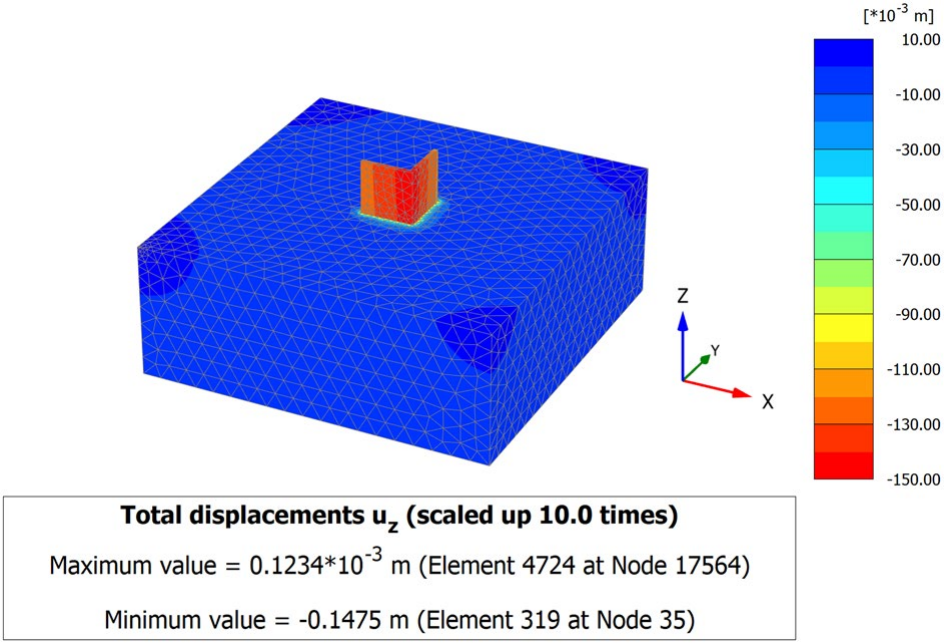
3D model of the maximum vertical displacement Uz of historical buildings in the Najran area.

**Figure 15:**
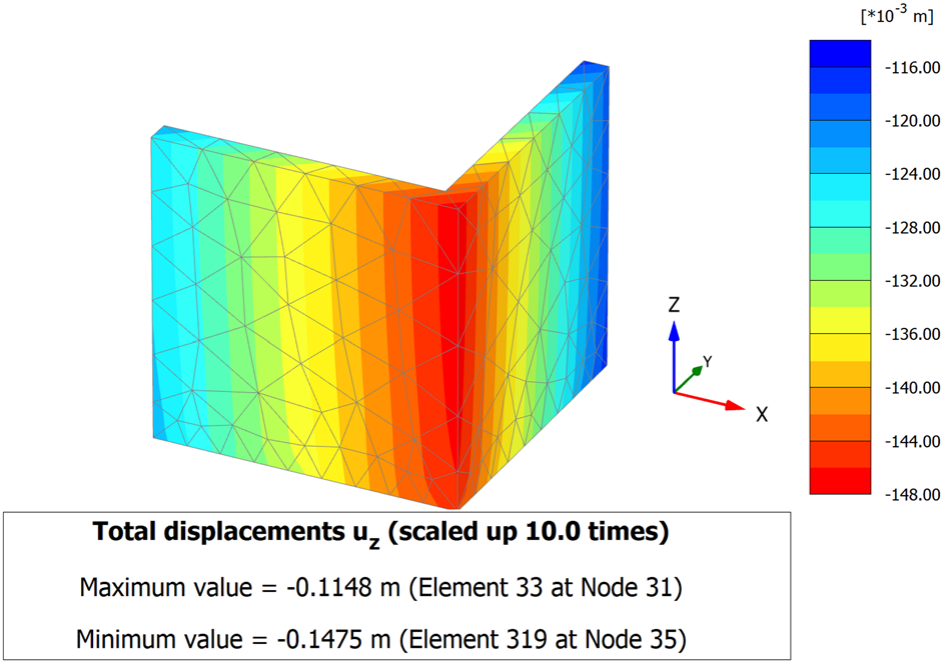
3D model of the total displacement of the walls shows the pattern of settlement of the wall Uz of a historical building in the Najran area.

**Figure 16:**
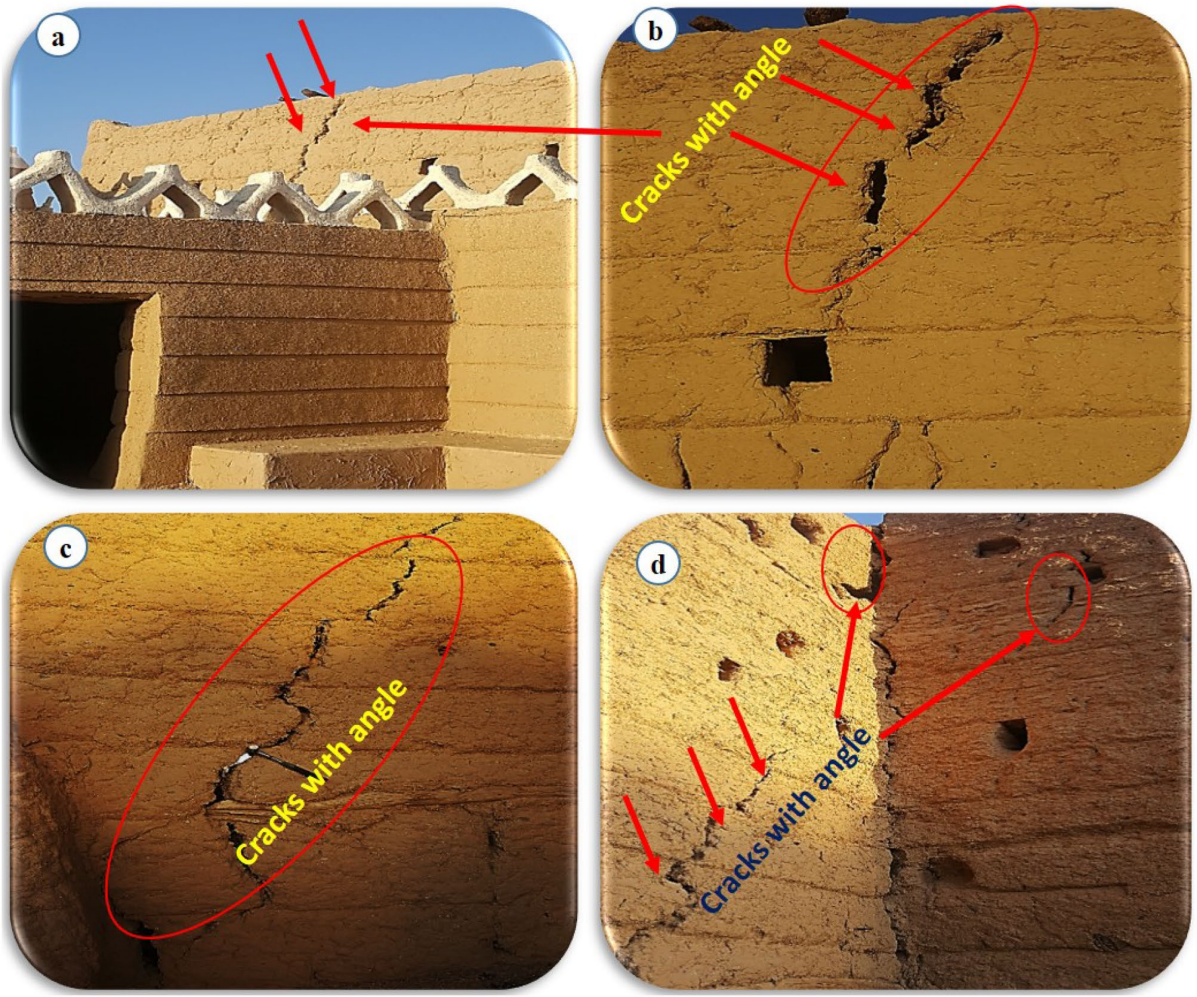
Site photographs illustrating the real cracks and their directions in the walls of an unmaintained old historical building.

**Figure 17:**
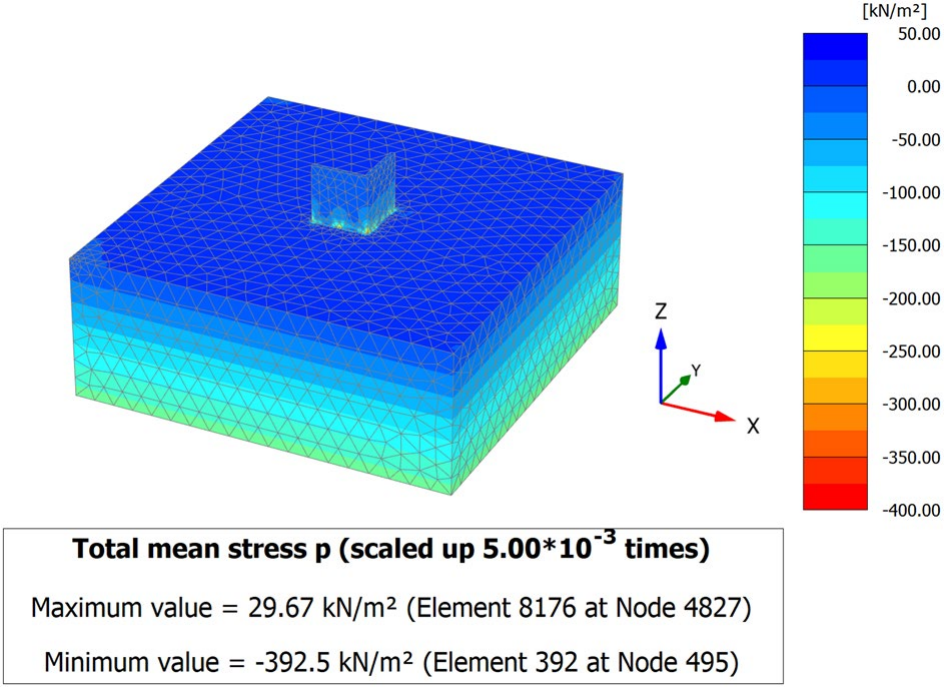
3D model of the total and maximum mean stress of historical buildings in the Najran area.

**Figure 18:**
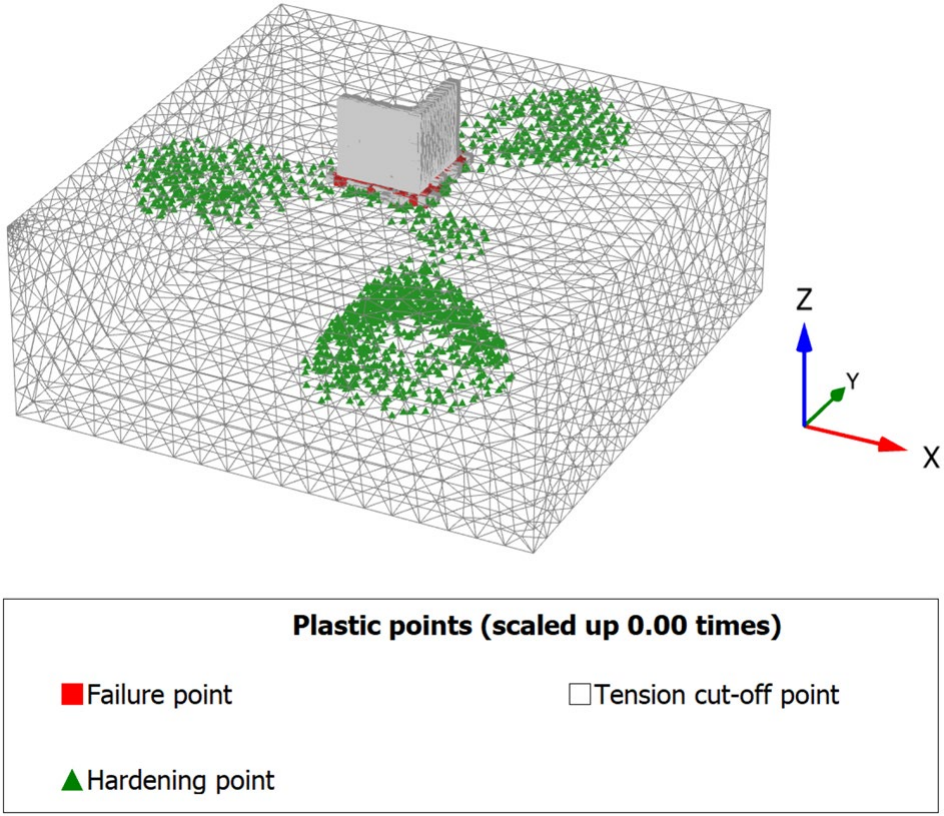
3D model of tension cut-off points and failure of historical buildings in the Najran area.

When a mathematical analytical model is used correctly, it can provide information regarding the characteristics, degree, and position of damage, as well as problematic zones and safety levels. Figure 12 illustrates the geometry and finite element discretization of the 3D model and the deformed mesh. The computed total displacement |U| and vertical displacement Uz of the silty sand soil layer with an earthen structural loading, which appears dispersed under the superstructure of the heritage building, are presented in Figs. 13 and 14, respectively. The total value of vertical displacement is 0.1521 m. Figure 15 shows the maximum and minimum settlement of the simulated earthen walls because of the settlement of the silty sand layer under the loading of the self-weight of the walls as well as the distributed dead and live loads. Based on the results in Fig. 15, one can note that the pattern of the wall settlement is inclined at an angle that looks like the angle of cracks seen in the photo presented in Fig. 16 for the cracked old earthen building. This can lead to the conclusion that the reasons behind the failure of the old earthen building structures (that are not maintained periodically) are excessive settlement and environmental conditions. Sometimes, due to the intensity of rainfall in the area, the foundations and the soil layer below them are saturated, and thereafter they begin to dry, which generates subsidence and cracks in the walls of the earthen building since they are the bearing elements in the building structure. Figure 17 shows the maximum mean stress under the simulated historical structure and the subsurface soil, whereas Fig. 18 shows the tension cut-off points on the walls, covering the whole wall. This enhances the discussion stated above.

## Conclusions

The experimental portion of the study, concentrating on the sampling of specimens of the earthen masonry wall units and the determination of their strength, water absorption, micro-characterization, and evaluation of tests, has led to the formulation of the following conclusions:

- The findings indicate that Illite is a major ingredient. The identity of Illite is confirmed by EDX analysis, and the feldspars constitute about 53.4% by weight of the samples.
- Because masonry qualities vary, data around its mechanical properties must be gathered through testing; masonry strength identification may thus play a crucial role in the appraisal of historical and other present structures.
- The water absorption results showed that absorption is within the acceptable range, although it is close to the upper allowed limit.

To avoid the possible successive appearance of cracks and masonry deterioration, any major increase in the loading of the existing heritage masonry structure, as well as any pertaining interventions or improvements, must be subjected to a comprehensive qualitative evaluation, or enough input values should be available for an accurate numerical assessment. Thus, in the numerical part of the paper, the model of an earthen heritage building is developed. Based on the modeling results, the following could be drawn:

- The maximum value of vertical settlement Uz is 0.1475 m.
- The maximum settlement of the simulated earthen walls is because of the settlement of the silty sand layer under the loading of the self-weight of the walls as well as the distributed dead and live loads of the whole building.
- The reasons behind the failure of the old earthen building structures (that are not maintained periodically) are excessive settlement and environmental conditions effects.
- The pattern of the wall’s settlement is inclined at an angle, which looks like the angle of cracks observed in the reality of unmaintained old historical buildings in the study area.
- As a result of surrounding environments, wind velocity, rain, sun rays, and man-made activation are the most common reasons for building cracking, especially in the lower areas.
- Since it is so difficult to build a model, apply real conditions, and monitor a historical building in the lab to verify the simulation outcomes, field observations of many historical buildings in the Najran area were used to confirm the simulation results.

Finally, the integrated geotechnical and numerical models could provide insights for assessing geoenvironmental risks, identifying the key deterioration causes in historic buildings, and providing an understanding of material qualities and failure causes not only in the study area but also in other similar places around the world.

## Data Availability

The data used in this manuscript can be obtained from the corresponding author upon reasonable request.
